# Effects of some anti-diabetic herbal extracts on the insulin-degrading enzyme in human colon cancer Caco-2 cell line

**DOI:** 10.22038/AJP.2022.19982

**Published:** 2022

**Authors:** Mahtab Norouzi, Hossein Saghi, Reza Mohebbati, Farshad Mirzavi, Amir Reza Afshari, Mohammad Soukhtanloo

**Affiliations:** 1 *Department of Clinical Biochemistry, Faculty of Medicine, Mashhad University of Medical Sciences, Mashhad, Iran*; 2 *Student Research Committee, Faculty of Medicine, Mashhad University of Medical Sciences, Mashhad, Iran*; 3 *Department of Physiology, Faculty of Medicine, Gonabad University of Medical Sciences, Gonabad, Iran*; 4 *Applied Biomedical Research Center, Mashhad University of Medical Sciences, Mashhad, Iran*; 5 *Department of Physiology and Pharmacology, Faculty of Medicine, North Khorasan University of Medical Sciences, Bojnurd, Iran*; 6 *Pharmacological Research Center of Medicinal Plants, School of Medicine, Mashhad University of Medical Sciences, Mashhad, Iran*

**Keywords:** Insulin-degrading enzyme Phaseolus vulgaris, Allium cepa, Portulaca oleracea, Cinnamomum verum, Citrullus colocynthis

## Abstract

**Objective:**

Type 2 diabetes mellitus (T2DM) is a condition characterized by insufficient insulin production or insulin resistance. The insulin-degrading enzyme (IDE) is responsible for degrading insulin and is a potential drug target for T2DM treatment. Numerous activities have been proposed for plant extracts, but research on the effects of plant extracts on IDE expression and activity is riddled with drawbacks.

**Materials and Methods:**

We investigated the effect of *Phaseolus vulgaris, Allium cepa, Portulaca oleracea, Cinnamomum verum, *and* Citrullus colocynthis* extracts on the expression and activity of IDE in the Caco-2 cell line.

**Results:**

Findings of RT-PCR showed that IDE gene expression was reduced following treatment with *P. vulgaris, C. colocynthis, *and* C. verum* extracts. The results of IDE activity with fluorogenic peptide substrate V also indicated that *P. vulgaris, C. colocynthis, *and* P. oleracea* extracts reduced IDE activity in a significant and dose-dependent manner.

**Conclusion:**

The hydroalcoholic extracts studied, except for *A.* *cepa*, can prevent insulin degradation by reducing the expression and activity of the IDE enzyme. This new insight into the effects of herbal medicines on IDE activity can help future studies.

## Introduction

Diabetes mellitus (DM) is the ninth leading cause of death worldwide. The incidence rate of DM is growing daily, and it is estimated to reach around 642 million by 2040 (Hussain and Ali, 2016 ▶). Approximately 90% of all patients are known type 2 diabetes mellitus (T2DM) cases, and about 4.6 million people lost their lives due to T2DM in 2011 (Hussain and Ali, 2016 ▶). 

T2DM arises from unacceptable insulin levels and/or improper responses to this hormone that controls blood sugar levels. Reducing the clearance of insulin could be a practical approach to ameliorate insulin levels in these patients. This is where a group of inhibitors called insulin-degrading enzyme (IDE) inhibitors come into action. IDE is a zinc metalloprotease that expresses ubiquitously. As the name implies, IDE binds to insulin with high affinity and inactivates this hormone through its degradation. Apart from insulin, other bioactive peptides such as glucagon, amylin, and amyloid-beta (Aβ) are targets of IDE (Tang, 2016 ▶). Maianti et al. showed for the first time that an IDE inhibitor could increase insulin's action in rabbits and rats (Maianti et al., 2014 ▶). This finding strengthened the potential use of IDE inhibitors to improve the effectiveness of insulin. 

Although some IDE inhibitors such as Ii1, BDM41367, 6bk, BDM44768, and NTE-1 have been discovered to improve glucose tolerance, the multi-functional property of IDE has restricted the application of inhibitors in clinical approaches (Deprez-Poulain et al., 2015 ▶; Leissring et al., 2010 ▶; Shen et al., 2006 ▶). For example, given the impact of IDE on the clearance of inflammatory chemokines, its inhibition could be coupled with the propagation of inflammatory diseases such as atherosclerosis (Caravaggio et al., 2013 ▶). Given the importance of IDE suppression in T2DM treatment, it seems that further studies are essential to find a product that could suppress IDE without inducing any harmful effects (Steneberg et al., 2013 ▶). Natural compounds such as those which could be derived from plants, appear to be ideal sources for identifying agents with IDE suppressive properties as they could reduce the clearance of insulin without impacting this enzyme’s other substrates such as glucagon and, more importantly, without any unfavorable side effects. Accordingly, this study evaluated the effects of five extracts, including the hydroalcoholic extracts of *Phaseolus vulgaris* (HEPV), *Citrullus colocynthis* (HECC), *Portulaca oleracea* (HEPO), *Allium cepa* (HEA. cepa), and *Cinnamomum verum* (HAEC) on IDE expression and activity in Caco-2 cells (Bai et al., 1995 ▶).

## Materials and Methods

**Reagents**

The RPMI 1640 culture medium and DMEM High Glucose were obtained from Gibco (Grand Island, NY, USA). Penicillin-streptomycin, fetal calf serum (FCS), and fluorogenic peptide substrate V were purchased from PAN Biotech (Germany), Invitrogen (Iran), and RandD Systems (USA), respectively. The 3-(4, 5-dimethylthiazol-2-yl)-2, 5-diphenyl-2H-tetrazolium bromide (MTT) powder and dimethyl sulfoxide (DMSO) were bought from Sigma-Aldrich (St. Louis, MO, USA).

**Plants and extracts**

*P. vulgaris, C. colocynthis, P. oleracea, A. cepa*, and *C. verum* whole plants were provided by the Department of Pharmacology, Faculty of Medicine, Mashhad University of Medical Sciences, Mashhad, Iran. *P. oleracea*, *A. cepa *and* P. vulgaris* were identified by Herbarium center of Ferdowsi University and voucher samples were preserved for reference in the herbarium of Faculty of Agriculture (Voucher No. of *P. oleracea*, *A. cepa *and* P. vulgaris* were 2240-1615-12, E1130 and E1028-FUMH, respectively). *C. colocynthis* fruits were authenticated by an expert botanist. A voucher specimen was deposited (No. 484) in herbarium of the Payam Noor University, Dargaz, Iran. The dried barks of *C. verum* were identified in the herbarium, department of pharmacognosy, School of Pharmacy, Mashhad University of Medical Sciences, Mashhad, Iran.

**Preparation of extracts**

After washing and drying them, plants were extracted by the Soxhlet device with ethanol 70% as the solvent (Shafiee-Nick et al., 2012 ▶). 

According to our protocol, all herbs were weighted and subjected to extraction with 70% ethanol in a Soxhlet apparatus for 72 hr. The hydro-alcoholic extract was then dried and crude extract was kept frozen at below −18^◦^C for the following use. The yield of extraction was calculated. The result of yield of extracts is shown in Table 1.

**Table 1:** The percentage yield of crude extract of the plants after the extraction by Soxhlet method

***Phaseolus*** ***Vulgaris***	***Citrull*** ***us*** ***colocynthis***	***Portulaca*** ***oleracea***	***Cinnamomum*** ***verum***	***Allium cepa***	**The plant **
50	50	150	50	50	**Weight (g)**
13.71	11.85	13.55	15	24.4	**Weight after Soxhlet (g)**
27.42	23.7	9	30	48.8	**Yield of crude extract (%)**

The yield of *A.cepa* extract was greater than the other plants and the yield of *P. oleracea* extract was the lowest among the plants.

**Standardization of the extracts of** ***P. vulgaris*** **,** ***C. colocynthis*** **,** ***P. oleracea*** **,** ***A. cepa*** **, and** ***C. verum***

The extracts of* P. vulgaris, C. colocynthis, P. oleracea, A. cepa*, and *C. verum* were standardized based on the content of phenolic compounds. A sample of 20 µl of the plant extract (10 mg/ml) or gallic acid as the standard (50-500 mg/L) was mixed with 100 µl of the Folin-Ciocalteu reagent and 300 µl of sodium carbonate solution (1 mol/L). The volume of the mixture was adjusted to 2 ml with deionized water. After 2 hr, absorbance was measured at 765 nm by a spectrometer. The standard curve was drawn for gallic acid (y = 0.0008x + 0.0223, R2 = 0.982) and the extract's phenolic compound content was calculated as milligram of gallic acid equivalent (Hooshmand et al., 2021 ▶).

**Cell cultures**

The human colorectal adenocarcinoma cell line Caco-2 (NCBI code C139) was purchased from the National Cell Bank of Iran (NCBI), Pasteur Institute (Tehran, Iran) and cultured in 50% RPMI 1640+35% DMEM High Glucose with 10% FBS and antibiotics (100 μg/ml streptomycin and 100 U/ml penicillin) (Farsani et al., 2018 ▶).

**Cell proliferation assay**

Cell proliferation was assessed by the colorimetric MTT assay (Zamiri-Akhlaghi, Rakhshandeh, Tayarani-Najaran, and Mousavi, 2011 ▶). In brief, Caco-2 cells were seeded in 96 wells (8×10^3^/well) and kept overnight. The cells were incubated with various concentrations of HEPV (16000, 8000, 4000, 2000, 1000, 500, 250, 125, 62.5, 31.25, and 15.6 µg/ml), HECC (4000, 2000, 1000, 500, 250, 125, 62.5, 31.25, and 15.6 µg/ml), HEPO (1600, 800, 400, 200, 100, 50, 25, and 12.5 µg/ml), HEA. cepa (4000, 2000, 1000, 500, 250, and 125 µg/ml), and HAEC (1000, 500, 250, 125, 62.5, 31.25, and 15.6 µg/ml) for 24 hr. Then, 10 µl of the MTT solution (phosphate buffer, 5 mg/ml) was added to each well. After 3-4 hr, the formazan residue was dissolved in DMSO. The absorbance at 545 and 630 nm (background) was measured on a Stat FAX303 plate reader. All the treatments were performed in triplicate.

**RNA analysis and quantitative reverse transcription- (qRT-) PCR**

Total RNA was extracted from the cells treated with HEPV (600, 300, 150, and 75 µg/ml), HECC (100, 50, and 25 µg/ml), HEPO (200, 100 and 25 µg/ml), HEA. cepa (600, 300, and 150 µg/ml), and HAEC (40, 20, and 10 µg/ml) according to the manufacturer’s instructions (Yekta Tajhiz, Tehran, Iran). Subsequently, RNAs were reverse-transcribed using the cDNA synthesis kit (Parstoos, Iran) using the Light-Cycler 96 real-time PCR system (Roche Applied Science, USA). The quantitative RT-PCR analysis was performed by RealQ Plus 2X Master Mix Green-without Rox™ (Amplicon, Denmark). Next, a quantitative RT-PCR was carried out with specific primers for GAPDH and IDE enzymes. The 2^−ΔΔCt^ method was adopted to analyze the relative expression of target genes. The primer sequences (forward and reverse) are listed in Table 2.

**Table 2:** List of primers used in the qRT-PCR analysis

**Gene symbol**	**Gene name**	**Primers (5ʹ → 3ʹ)**	**Accession number**
**IDE**	Insulin-degrading Enzyme	Forward: GGAACCTTGCTTCAACACCCTG	NM_001322797
		Reverse: AGCCCTGTATGCCATTAGCTCG	
**GAPDH**	Glyceraldehyde-3-phosphate dehydrogenase	Forward: CTGGGCTACACTGAGCACC	NM_002046.7
		Reverse: AAGTGGTCGTTGAGGGCAATG	

**Fluorogenic assay of enzyme activity**

Fluorogenic peptide substrate V (7-methoxycoumarin-4-yl-acetyl-RPPGFSAFK-2, 4-dinitrophenyl) obtained from RandD Systems, was used as the substrate for this enzyme (Catalogue Number: ES005). Cell lysates at a concentration of 10 µM were incubated with protease inhibitor cocktail 1x (cell Signaling) at 37°C for 15 min to inhibit other peptidases, and then a certain amount of working solution (Substrate V 10 µM, Tris 50 Mm, NaCl 1M (pH 7.5)) was added to each well (final volume in each well: 100 µL). The microplate was then incubated in an incubator at 37°C for 30 min. The Perkin Elmer fluorescent plate reader measured enzyme activity with excitation at 320 nm and emission at 405 nm.

**Statistical analysis**

The experimental data were analyzed using GraphPad Prism (GraphPad 7, San Diego, CA, USA). The data normality was checked using Kolmogorov–Smirnov distribution test. The statistical difference between groups was assessed using a one-way analysis of variance (ANOVA) followed by Bonferroni's test. The data are presented as mean±standard error of the mean, and a p<0.05 was considered to indicate a statistically significant difference. All the data were examined in triplicate against untreated control cells and collected from three ‎independent experiments.

## Results

**Phenolic content of in** ***P. vulgaris*, *C. colocynthis*, *P. oleracea*, *A. cepa*, and *C. verum* extract **

The content of total phenols in the extract of *A. cepa, C. colocynthis, P. vulgaris, P. oleracea and C. verum* was 28, 40, 22, 57, and 372 mg gallic acid equivalent per gram of the crude extract, respectively.

**The hydroalcoholic extracts inhibited the proliferation of Caco-2 cells**

**Figure 1:**
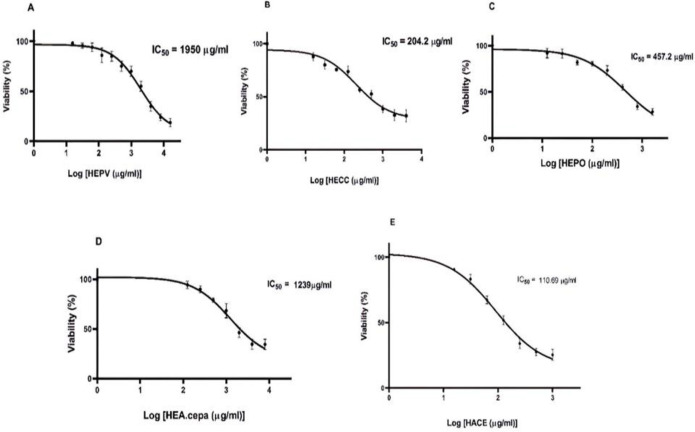
The MTT assay determined cell proliferation. A: Dose-dependent effects of HEPV on cell viability in Caco-2 cells following 24-hr treatment. B: Dose-dependent effects of HECC on cell viability in Caco-2 cells following 24-hr treatment. C: Dose-dependent effects of HEPO on cell viability in Caco-2 cells following 24-hr treatment. D: Dose-dependent effects of HEA. cepa on cell viability in Caco-2 cells following 24-hr treatment. E: Dose-dependent effects of HACE on cell viability in Caco-2 cells following 24-hr treatment. **p<0.01 and ***p<0.001 versus the control group. Data are presented as the mean±standard error of the mean (n=3)

To assess whether hydroalcoholic extracts of the five compounds could reduce the proliferative capacity of Caco-2 cells, the cells were treated with relevant concentrations of each extract for 24 hr, and their metabolic activity was assessed using the MTT assay. As presented in Figure 1, all the extracts could diminish the metabolic activity of Caco-2 cells in a dose-dependent manner. The IC50 for HEPV, HECC, HEPO, HEA. Cepa, and HACE in Caco-2 cells was 1950, 204.2, 457.2, 1239, and 110.69 µg/ml, respectively.

**The effect of** **hydroalcoholic extracts** **on IDE expression**

**Figure 2:**
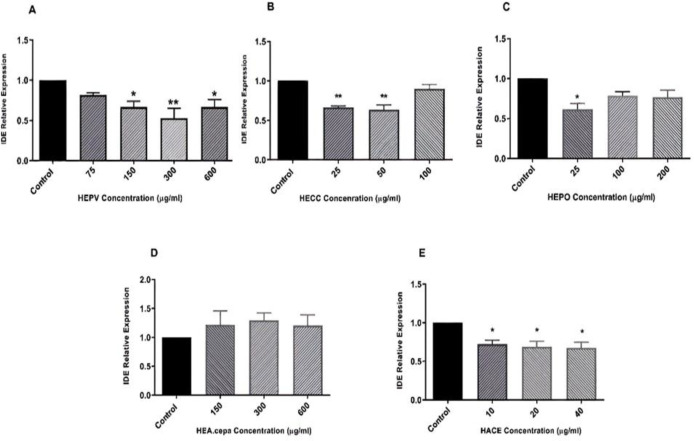
Relative IDE expression induced by hydroalcoholic extracts after 24-hr treatment. Caco-2 Cells were treated with HEPV ((Fig. 2A) 75, 150, 300 and 600 µg/ml), HECC ((Fig. 2B) 25, 50 and 100 µg/ml), HEPO ((Figure 2C) 25, 100 and 200 µg/ml), HEA. cepa ((Figure 2D) 150, 300 and 600 µg/ml), and HAEC ((Figure 2E) 10, 20 and 40 µg/ml) for 24 hr, and then, the expression of IDE was evaluated by qRT-PCR analysis (*p<0.05 and **p<0.001 compared with the control)

The qRT-PCR analysis was performed to evaluate the expression of IDE upon treatment of Caco-2 cells with different concentrations of the extracts. As depicted in Figure 2, while HEPV and HECC could significantly reduce the expression of IDE (p<0.05 and p<0.001), other extracts failed to alter the expression of this enzyme in Caco-2 cells. Note that higher concentrations of HEPV and HECC could increase the expression on IDE. 

**The effect of hydroalcoholic extracts on IDE activity**

**Figure 3:**
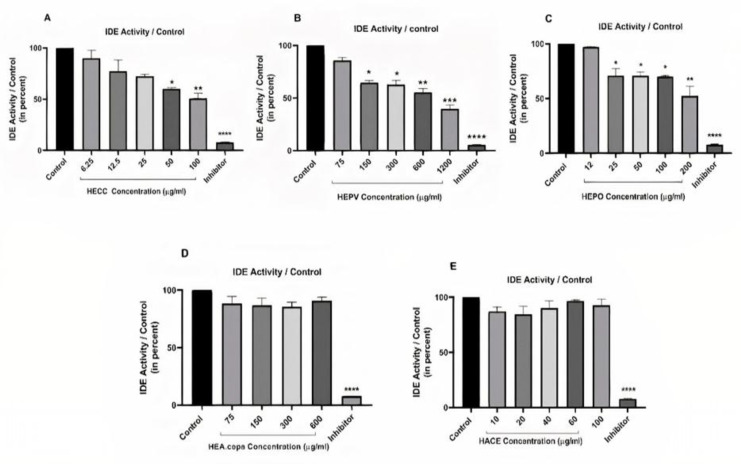
The effect of hydroalcoholic extracts and EDTA (1 mM) on the activity of IDE in Caco-2 cells (24 hr). Our results show that while HECC (Figure 3A), HEPV (Figure 3B), and HEPO (Figure 3C) could diminish the activity of the enzyme, HEA. cepa (Figure 3D) and HAEC (Figure 3E) did not have any effects on the activity of IDE (*p<0.05, **p<0.001, and ***p<0.001 as compared with the control)(n=3)

We examined the effect of HEPV, HECC, HEPO, HEA. Cepa, and HAEC on IDE enzymatic activity in Caco-2 Cells by a fluorogenic assay. EDTA (1 mM (was used as a positive control to inhibit IDE activity. Our results indicated that, compared to EDTA which could significantly reduce the expression of IDE, only HEPV, HECC, and HEPO could diminish the enzyme's activity upon 24 hr treatment (p<0.05, p<0.001, and p<0.001). HAEC and HEA. Cepa did not have any considerable influence on IDE activity (Figure 3).

## Discussion

Following the first description of T2DM, several valuable therapeutic approaches have been developed to increase the survival of the patients (Olokoba et al., 2012 ▶). Perhaps T2DM is not fatal by itself; however, its association with 

other devastating diseases such as cardiovascular diseases, Alzheimer’s, and cancer turned this disease into the most serious health crisis of the 21^st^ century (Barbagallo and Dominguez, 2014 ▶; Hu et al., 2002 ▶; Satija et al., 2015 ▶). Thus, the list of drugs to treat this disease is growing daily; among them, IDE inhibitors have enjoyed unprecedented success in T2DM treatment (Tang, 2016 ▶). IDE inhibitors target a neutral Zn^2+^-metalloendopeptidase that participates in the intracellular process of insulin (González-Casimiro et al., 2021 ▶). The results of previous studies declared that this group of inhibitors could even counteract insulin resistance in T2DM patients. Despite their valuable efficacy, the hurdles in the design of the inhibitors have diminished the interest in their clinical applications (Leissring et al., 2021 ▶). Before 2010 when selective IDE inhibitors were developed (Leissring et al., 2010 ▶), compelling lines of studies used non-specific inhibitors of IDE such as zinc-chelators, thiol-alkylating compounds, and the cyclic peptide bacitracin to reduce the activity of IDE (Leissring et al., 2021 ▶). Moreover, numerous herbal and natural compounds have been identified to alter insulin clearance in diabetic models (Brandimarti et al., 2013 ▶; Kim et al., 2019 ▶). Herein, we assessed the efficacy of five herbal extracts derived from *P. vulgaris, C. colocynthis, P. oleracea, A. cepa*, and *C. verum* on the activity and expression of IDE in Caco-2 cells. 

The results indicated that all the tested extracts possess anti-proliferative effects, as revealed by the significant reduction in the metabolic activity of Caco-2 cells in a dose-dependent manner. In agreement with our results, Hwang et al. have suggested that the extract derived from *A. cepa* could halt the proliferation capacity of adipocytes by downregulating the expression of fatty acid synthesis (Hwang et al., 2012 ▶). In another study, Kwon et al. reported that the *C.* *verum* extract suppressed the nuclear activity of NF-κB, thereby inhibiting cell growth in melanoma cells (Kwon et al., 2010 ▶). It has also been suggested that the polyphenols in the *C. verum* extract could reduce the phosphorylation of insulin receptors in T2DM patients (Baker et al., 2008 ▶). The tight and reciprocal interplay between the regulation of glucose metabolism and cell proliferation has been well-reviewed in previous studies (Zhu and Thompson, 2019 ▶). It has been suggested that, through integrating with diverse signaling pathways such as PI3K/Akt axis, the excessive amount of insulin suppresses the expression of different cyclin kinase (CDK) inhibitors to reinforce the progression of the cell cycle (Hopkins et al., 2020 ▶). Okada et al. also suggested that upon insulin receptor activation, the FoxO1/Pdx-1 signaling pathway is activated in the pancreatic β-cells to compensate for cell growth and induce insulin resistance (Okada et al., 2007 ▶). Glucose and its related molecules could also activate autophagy flux within the cells, a mechanism that provides excessive energy for cell proliferation through degradation of unnecessary proteins and dysfunctional subcellular organelles (Ha et al., 2015 ▶). Given these, it is reasonable to assume that all the mentioned extracts in the present study could exert anti-proliferative effects by interacting with the critical molecules in regulating cell growth and glucose metabolism. 

Apart from its role in the degradation of insulin, the results of recent studies reflected another face for IDE, this time as a regulator of cell proliferation (Tundo et al., 2013 ▶). The interplay between retinoblastoma proteins (RB) and IDE has been suggested to play fundamental roles in cell growth regulation (Radulescu et al., 2010 ▶). Given these and based on the anti-proliferative capacity of the extracts, it was of particular interest to evaluate whether these extracts could diminish the expression of IDE in Caco-2 cells. Our results showed that among the five herbal extracts, the low concentrations of HEPV, HEPO, and HECC extracts derived from *P. vulgaris, P. oleracea, *and* C. colocynthis*, respectively, could effectively reduce the enzymatic activity of IDE in Caco-2 cells. More interestingly, HEPV and HECC were also influential in diminishing the expression of IDE in adenocarcinoma cells, suggesting that the anti-proliferative effects of the extracts could be mediated by IDE down-regulation. To the best of our knowledge, although studies have emphasized the anti-oxidant and hypolipidemic effects of HEPV on diabetic mice (Pari and Venkateswaran, 2003 ▶), no study has addressed the potential of HEPV in reducing IDE expression; thus, the current study presents for the first time that the anti-diabetic effect of the extracts is mediated through suppression of this enzyme. Note that various mechanisms are involved in IDE enzymatic activity, including the ability to cause chemical changes in cysteines in the IDE structure by IDE inhibitors or modulators. Researchers suggest that some compounds bind to cysteines in the enzyme and inhibit enzyme activity (Song et al., 2003 ▶). According to our study, HEPV effectively reduces the expression and activity of this enzyme; in addition to the mechanism of insulin secretion from pancreatic β cells, we propose the prevention of insulin degradation by interaction with amino acids at the catalytic site of the enzyme. In contrast to HEPV, the mechanism through which, both *C. colocynthis *and* P. oleracea *extracts could induce anti-diabetic effects has been well-established in previous studies. *C. colocynthis* seems to have a stimulatory impact on the expression of peroxisome proliferator-activated receptor-γ (PPAR-γ) (Jemai et al., 2020 ▶), a ligand-activated transcription factor belonging to the nuclear receptor with a fundamental role in glucose homeostasis (Cataldi et al., 2021 ▶). When PPAR-γ is activated, it can bind to the α receptor of retinoic acid 9-cis (RXRα) to change the expression of a wide range of downstream targets such as IDE (Du et al., 2009 ▶). Given these and based on our findings which indicated that HECC could reduce the expression of IDE in Caco-2 cells, it could be reasonable to assume that this extract might diminish the expression and activity of this enzyme through interacting with PPAR-γ. Another study found that interleukin (IL)-6 increased IDE expression and activity (Kurauti et al., 2017 ▶). The polysaccharides derived from *P. oleracea* extract have also shown suppressive effects on the expression of IL-6 and tumour necrosis factor (TNF)-α in diabetic rats (Bai et al., 2016 ▶). IL-6 is one of the main cytokines in inflammatory responses; however, it could reinforce the activity of metalloendopeptidase. It seems that this extract reduced the activity of IDE, at least in part, in an IL-6-dependent manner. Although HEPO did not alter the expression of IDE, it may have regulated post-translational changes without affecting mRNA. These cases should be further investigated in the future. 

The results demonstrated that the hydroalcoholic extracts of *C. verum* and *A. cepa* did not induce a significant change in the expression and activity of IDE. In 2019, the effects of orlistat, as well as *C. verum* as a natural lipase inhibitor on the management of obesity were investigated. This study reported that the significant reduction in insulin levels in *C. verum* therapy was not related to IDE activity. Similarly, our research showed that the treatment of cells with *C. verum* extract did not display a change in the expression or activity of IDE (Khedr et al., 2020 ▶). Based on research evidence, *A. cepa* extract stimulates cellular glucose uptake and hypoglycemia. Still, it is not clear whether cellular glucose uptake may be due to increased insulin secretion or decreased insulin degradation (Jevas, 2011 ▶). Jevas et al. concluded that this hypoglycemic activity arises from allylpropyldisulphide (APDS) in aqueous *A. cepa* extract. They speculated that APDS *inactivated* IDE in the short term, leading to increased insulin levels and decreased blood sugar (Jevas, 2011 ▶). The analogs of APDS targeted this enzyme. Among these analogues, two analogues showed hypoglycemic activity against this enzyme which, of course, had short-term effects (Mwenga, 2018 ▶). According to our results and those of other studies, more research should be conducted on different kinds of *A. cepa* extract and effective components to clarify this issue. 

In conclusion, our findings suggested for the first time that the hydroalcoholic extracts of *P. vulgaris*, *C. colocynthis*, and *P. oleracea* might have a suppressive impact on the expression and the activity of IDE. It could be postulated that these compounds could be used in the treatment of T2DM. However, further *in vitro* and *in vivo* studies are required to study the mechanism of action of these extracts more precisely. 

## Conflicts of interest

The authors have declared that there is no conflict of interest.

## References

[B1] Bai JP, Hsu MJ, Shier WT (1995). Insulin-degrading enzyme in a human colon adenocarcinoma cell line (Caco-2). Pharm Res.

[B2] Bai Y, Zang X, Ma J, Xu G (2016). Anti-diabetic effect of Portulaca oleracea Polysaccharideandits mechanism in diabetic rats. Int J Mol Sci.

[B3] Baker WL, Gutierrez-Williams G, White CM, Kluger J, Coleman CI (2008). Effect of cinnamon on glucose control and lipid parameters. Diabetes Care.

[B4] Barbagallo M, Dominguez LJ (2014). Type 2 diabetes mellitus and Alzheimer’s disease. World J Diabetes.

[B5] Brandimarti P, Costa-Júnior J, Ferreira S, Protzek A, Santos G, Carneiro E, Boschero A, Rezende L (2013). Cafeteria diet inhibits insulin clearance by reduced insulin-degrading enzyme expression and mRNA splicing. J Endocrinol.

[B6] Caravaggio JW, Hasu M, Maclaren R, Thabet M, Raizman JE, Veinot JP, Marcel YL, Milne RW, Whitman SC (2013). Insulin-degrading enzyme deficiency in bone marrow cells increases atherosclerosis in LDL receptor-deficient mice. Cardiovascular Pathol.

[B7] Cataldi S, Costa V, Ciccodicola A, Aprile M (2021). PPARγ and diabetes: Beyond the genome and towards personalized medicine. Curr Diabetes Rep.

[B8] Deprez-Poulain R, Hennuyer N, Bosc D, Liang WG, Enée E, Marechal X, Charton J, Totobenazara J, Berte G, Jahklal J (2015). Catalytic site inhibition of insulin-degrading enzyme by a small molecule induces glucose intolerance in mice. Nat Commun.

[B9] Du J, Zhang L, Liu S, Zhang C, Huang X, Li J, Zhao N, Wang Z (2009). PPARγ transcriptionally regulates the expression of insulin-degrading enzyme in primary neurons. Biochem Biophys Res Commun.

[B10] Farsani TM, Motevaseli E, Neyazi N, Khorramizadeh MR, Zafarvahedian E, Ghahremani MH (2018). Effect of passage number and culture time on the expression and activity of insulin-degrading enzyme Caco-2 Cells. Iran Biomed J.

[B11] González-Casimiro CM, Merino B, Casanueva-Álvarez E, Postigo-Casado T, Cámara-Torres P, Fernández-Díaz CM, Leissring MA, Cózar-Castellano I, Perdomo G (2021). Modulation of insulin sensitivity by insulin-degrading enzyme. Biomed.

[B12] Ha J, Guan K-L, Kim J (2015). AMPK and autophagy in glucose/glycogen metabolism. Mol Aspects Med.

[B13] Hooshmand S, Mahdinezhad MR, Taraz Jamshidi S, Soukhtanloo M, Mirzavi F, Iranshahi M, Hasanpour M, Ghorbani A (2021). Morus nigra L. extract prolongs survival of rats with hepatocellular carcinoma. Phytother Res.

[B14] Hopkins BD, Goncalves MD, Cantley LC (2020). Insulin–PI3K signalling: an evolutionarily insulated metabolic driver of cancer. Nat Rev Endocrinol.

[B15] Hu FB, Stampfer MJ, Haffner SM, Solomon CG, Willett WC, Manson JE (2002). Elevated risk of cardiovascular disease prior to clinical diagnosis of type 2 diabetes. Diabetes Care.

[B16] Hussain A, Ali I (2016). Diabetes mellitus in Pakistan: A major public health concern. Arch Pharm Pract.

[B17] Hwang CK, Wagley Y, Law PY, Wei LN, Loh HH (2012). MicroRNAs in opioid pharmacology. J Neuroimmune Pharmacol.

[B18] Jemai R, Drira R, Makni M, Fetoui H, Sakamoto K (2020). Colocynth (Citrullus colocynthis) seed extracts attenuate adipogenesis by down-regulating PPARγ/SREBP-1c and C/EBPα in 3T3-L1 cells. Food Biosci.

[B19] Jevas C (2011). Anti-diabetic effects of Allium cepa (onions) aqueous extracts on alloxan-induced diabetic Rattus novergicus. J Med Plants Res.

[B20] Khedr NF, Ebeid AM, Khalil RM (2020). New insights into weight management by orlistat in comparison with cinnamon as a natural lipase inhibitor. Endocr.

[B21] Kim Y, Rouse M, González-Mariscal I, Egan JM, O’connell JF (2019). Dietary curcumin enhances insulin clearance in diet-induced obese mice via regulation of hepatic PI3K-AKT axis and IDE, and preservation of islet integrity. Nutr Metabol.

[B22] Kurauti MA, Costa-Júnior JM, Ferreira SM, Santos GJ, Sponton CH, Carneiro EM, Telles GD, Chacon-Mikahil MP, Cavaglieri CR, Rezende LF (2017). Interleukin-6 increases the expression and activity of insulin-degrading enzyme. Sci Rep.

[B23] Kwon H-K, Hwang J-S, So J-S, Lee C-G, Sahoo A, Ryu J-H, Jeon WK, Ko BS, Im C-R, Lee SH (2010). Cinnamon extract induces tumor cell death through inhibition of NFκB and AP1. BMC Cancer.

[B24] Leissring MA, González-Casimiro CM, Merino B, Suire CN, Perdomo G (2021). Targeting insulin-degrading enzyme in insulin clearance. Int J Mol Sci.

[B25] Leissring MA, Malito E, Hedouin S, Reinstatler L, Sahara T, Abdul-Hay SO, Choudhry S, Maharvi GM, Fauq AH, Huzarska M (2010). Designed inhibitors of insulin-degrading enzyme regulate the catabolism and activity of insulin. PloS One.

[B26] Maianti JP, Mcfedries A, Foda ZH, Kleiner RE, Du XQ, Leissring MA, Tang W-J, Charron MJ, Seeliger MA, Saghatelian A (2014). Anti-diabetic activity of insulin-degrading enzyme inhibitors mediated by multiple hormones. Nature.

[B27] Mwenga SA (2018). Synthesis and antiglycemic activity of in silico designed analogues of allylpropyldisulphide. University of Nairobi.

[B28] Okada T, Liew CW, Hu J, Hinault C, Michael MD, Kr̈Tzfeldt J, Yin C, Holzenberger M, Stoffel M, Kulkarni RN (2007). Insulin receptors in β-cells are critical for islet compensatory growth response to insulin resistance. Proceed Nat Acad Sci.

[B29] Olokoba AB, Obateru OA, Olokoba LB (2012). Type 2 diabetes mellitus: a review of current trends. Oman Med J.

[B30] Pari L, Venkateswaran S (2003). Effect of an aqueous extract of Phaseolus vulgaris on plasma insulin and hepatic key enzymes of glucose metabolism in experimental diabetes. Die Pharmazie-An Inte J Pharm Sci.

[B31] Radulescu RT, Duckworth WC, Levy JL, Fawcett J (2010). Retinoblastoma protein co-purifies with proteasomal insulin-degrading enzyme: implications for cell proliferation control. Biochem Biophys Res Commun.

[B32] Satija A, Spiegelman D, Giovannucci E, Hu FB (2015). Type 2 diabetes and risk of cancer. BMJ.

[B33] Shafiee-Nick R, Ghorbani A, Vafaee Bagheri F, Rakhshandeh H (2012). Chronic administration of a combination of six herbs inhibits the progression of hyperglycemia and decreases serum lipids and aspartate amino transferase activity in diabetic rats. Adv Pharmacol Sci.

[B34] Shen Y, Joachimiak A, Rosner MR, Tang W-J (2006). Structures of human insulin-degrading enzyme reveal a new substrate recognition mechanism. Nature.

[B35] Song E-S, Juliano MA, Juliano L, Hersh LB (2003). Substrate activation of insulin-degrading enzyme (insulysin): A potential target for drug development. J Biol Chem.

[B36] Steneberg P, Bernardo L, Edfalk S, Lundberg L, Backlund F, Östenson C-G, Edlund H (2013). The type 2 diabetes–associated gene Ide is required for insulin secretion and suppression of α-synuclein levels in β-cells. Diabetes.

[B37] Tang W-J (2016). Targeting insulin-degrading enzyme to treat type 2 diabetes mellitus. Trend Endocrinol Metabol.

[B38] Tundo GR, Sbardella D, Ciaccio C, Bianculli A, Orlandi A, Desimio MG, Arcuri G, Coletta M, Marini S (2013). Insulin-degrading enzyme (IDE): a novel heat shock-like protein. J Biol Chem.

[B39] Zamiri-Akhlaghi A, Rakhshandeh H, Tayarani-Najaran Z, Mousavi SH (2011). Study of cytotoxic properties of Rosa damascena extract in human cervix carcinoma cell line. Avicenna J Phytomed.

[B40] Zhu J, Thompson CB (2019). Metabolic regulation of cell growth and proliferation. Nat Rev Mol Cell Biol.

